# The Fur-like regulatory protein MAP3773c modulates key metabolic pathways in *Mycobacterium avium* subsp. *paratuberculosis* under in-vitro iron starvation

**DOI:** 10.1038/s41598-024-59691-3

**Published:** 2024-04-18

**Authors:** Sajani Thapa, Govardhan Rathnaiah, Denise K. Zinniel, Raul G. Barletta, John P. Bannantine, Marianne Huebner, Srinand Sreevatsan

**Affiliations:** 1 Department of Pathobiology and Diagnostic Investigation, College of Veterinary Medicine, Michigan State University, 784 Wilson Road, STEG300, East Lansing, MI 48824 USA; 2 School of Veterinary Medicine and Biomedical Sciences, University of Nebraska, Lincoln, USA; 3 National Animal Disease Center, Ames, IA USA; 4 Department of Statistics and Probability, Michigan State University, East Lansing, MI USA; 5 University Of Nebraska, Eppley Institute for Cancer Research, Lincoln, USA

**Keywords:** Computational biology and bioinformatics, Microbiology, Molecular biology, Pathogenesis

## Abstract

Johne’s disease (JD) is a chronic enteric infection of dairy cattle worldwide. *Mycobacterium avium* subsp. *paratuberculosis* (*MAP*), the causative agent of JD, is fastidious often requiring eight to sixteen weeks to produce colonies in culture—a major hurdle in the diagnosis and therefore in implementation of optimal JD control measures. A significant gap in knowledge is the comprehensive understanding of the metabolic networks deployed by *MAP* to regulate iron both in-vitro and in-vivo. The genome of MAP carries *MAP3773c, a* putative metal regulator, which is absent in all other mycobacteria. The role of *MAP3773c* in intracellular iron regulation is poorly understood. In the current study, a field isolate (K-10) and an in-frame *MAP3773c* deletion mutant (Δ*MAP3773c*) derived from K-10, were exposed to iron starvation for 5, 30, 60, and 90 min and RNA-Seq was performed. A comparison of transcriptional profiles between K-10 and Δ*MAP3773c* showed 425 differentially expressed genes (DEGs) at 30 min time post-iron restriction. Functional analysis of DEGs in Δ*MAP3773c* revealed that pantothenate (Pan) biosynthesis, polysaccharide biosynthesis and sugar metabolism genes were downregulated at 30 min post-iron starvation whereas ATP-binding cassette (ABC) type metal transporters, putative siderophore biosynthesis, PPE and PE family genes were upregulated. Pathway analysis revealed that the *MAP3773c* knockout has an impairment in Pan and Coenzyme A (CoA) biosynthesis pathways suggesting that the absence of those pathways likely affect overall metabolic processes and cellular functions, which have consequences on MAP survival and pathogenesis.

## Introduction

Johne’s disease (JD) is caused by *Mycobacterium avium* subsp. *paratuberculosis* (MAP). JD is a chronic subclinical enteropathy, causing progressive malnutrition and emaciation of affected animals. The herd level prevalence of JD was estimated at 70.4% by the United States (US) National Animal Health Monitoring System’s (NAHMS) Dairy 2007 study end. The true US dairy herd-level prevalence of MAP was redefined when a re-analysis using a Bayesian approach, at 91.1% ^2^ suggesting a greater impact on both agricultural economy and animal health. Furthermore, the disease poses economic burden to producers and infected animals through premature culling, diminished carcass value, reduced milk production and quarantine measures applied in the infected herds. In the US alone, the economic burden of MAP infection amounts to a loss of approximately US$198 million annually ^3^.

Iron is an essential cofactor in cellular enzymatic activity including electron transport, iron sulfur (Fe-S) cluster biogenesis, nucleic acid synthesis and oxidative stress response ^4^. It is now well-established that MAP requires iron supplementation for in-vitro laboratory culture ^5,6^. This presents a major hurdle in timely diagnosis and therefore implementation of optimal control measures. Thus, a deeper understanding of MAP iron physiology is an important first step in improving in-vitro culturing methods for MAP as well as gaining knowledge on its pathogenicity requirements.

In MAP, the iron dependent regulator (IdeR) is the primary transcriptional regulator of iron homeostasis. In the presence of iron, IdeR binds to a 19 bp promotor sequence known as “iron box” and represses the expression of genes involved in iron acquisition (*mbt*) and storage (*bfrA*) ^7^. In addition to IdeR, a native iron regulatory protein, the MAP genome contains a Fur-like transcriptional regulator, *MAP3773c* that is located on a genomic island or large genomic polymorphism (termed LSP 15) and is absent in other mycobacteria. LSP15 (5.4 kb) is predicted to encode several metal uptake systems, an ATP-binding cassette (ABC) transporter (*MAP3776c- MAP3774c*), a ferric uptake regulator (*MAP3773c*) and a cobalamin (vitamin B12) synthesis (*MAP3772c*) ^8^. LSP15 was likely acquired via horizontal gene transfer from an unrelated species and has been retained because it is expected to confer increased fitness under varying environments that the bacteria encounters during its lifecycle ^9^. *MAP3773c* encodes an ortholog of the ferric uptake regulator (Fur) that recognizes a 19 bp DNA promoter sequence motif (Fur box), and it is involved in metal homeostasis ^10,11^. Fur is well characterized in *Enterobacteriaceae* and has been shown to control iron metabolism, regulate defenses against oxidative stress, and is considered the master regulator of iron homeostasis ^12,13^. Further, the Fur protein operates as a repressor, by blocking RNA polymerase binding to the promoter region of genes involved in iron homeostasis repressing transcription ^14,15^ and in some situations can double up as an activator of gene expression in response to iron through indirect mechanism involving repression of small regulatory RNA ^16–18^. Furthermore, Fur has been shown to repress gene expression under iron-replete conditions and allow sufficient concentration of intracellular iron for essential iron-dependent metabolic pathways ^12,19,20^. *MAP3773c* has been recently confirmed as a metal regulator protein that recognizes many target sites in the genome either under iron-replete or deplete conditions. A recent ChIP-seq analysis identified several putative genomic binding sites upstream of genes that are likely involved in iron homeostasis in MAP ^11^.

A complete functional characterization of *MAP3773c* in response to iron availability is not yet established. In this study, the transcriptional regulation pathways of *MAP3773c* deployed by MAP to maintain iron homeostasis under iron restriction conditions was investigated. To elucidate the role of *MAP3773c* in response to iron starvation, a deletion of *MAP3773c* (Δ*MAP3773c*) was generated from a well characterized field isolate (MAP K10) through homologous recombination. Iron stress-related gene expression profiles were investigated in a time-course experiment to understand the temporal dynamics of iron regulation mechanism by *MAP3773c*. The early timepoints (5 min and 30 min post iron starvation) in the study were selected to capture the immediate response of the bacterium to iron limitation as the bacterium senses and responds to the abrupt decrease in iron availability. We considered the timing of iron restriction within the host cell context, which typically occurs within 30 min after the pathogens are phagocytosed. Furthermore, our study encompassed longer time points (60 min and 90 min post iron starvation) to capture the sustained and dynamic nature of the bacterial response over time. Thus, a comparative, time-course experimental design was applied to define transcriptional profiles of the parent and mutant strains and define critical metabolic pathways likely under the control of *MAP3773c*.

## Results

### Generation of a *MAP3773c* deletion mutant

**Figure 1:**
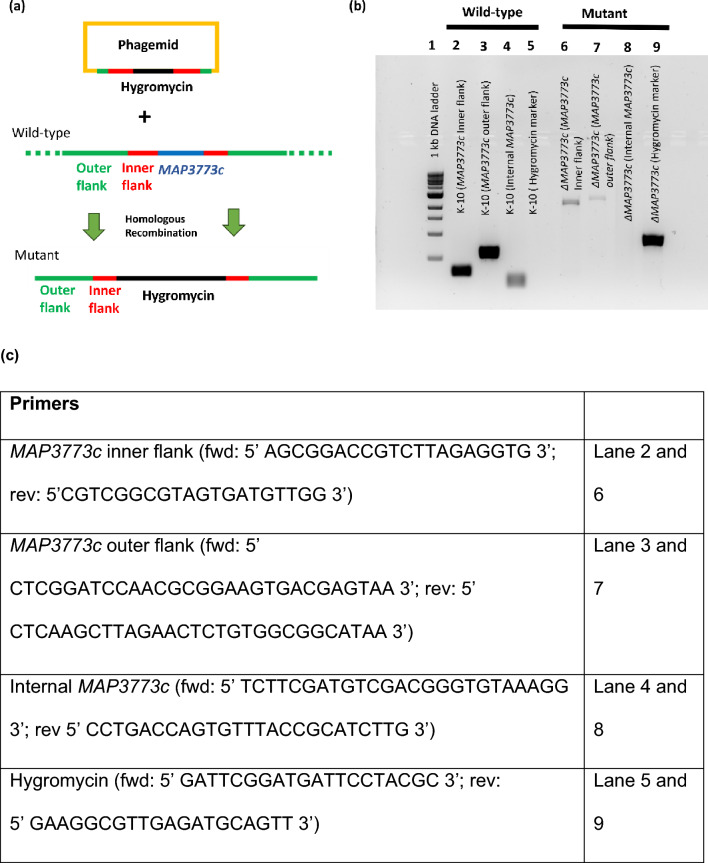
Generation of *MAP3773c* deletion mutant (*ΔMAP3773c*). (**a**) Schematic representation of the *MAP3773c* gene replaced by the hygromycin cassette in the MAP K-10 genome by homologous recombination between outer and inner flanking regions of *MAP3773c* of MAP K-10 via a phagemid, generating a deletion mutant. (**b**) 1% agarose gel confirming the presence of in-frame hygromycin resistance gene. Lane 1: 1 kb DNA ladder, Lane 2: MAP K-10 (*MAP3773c* inner flank), Lane 3: MAP K-10 (*MAP3773c* outer flank), Lane 4: MAP K-10 (internal *MAP3773c*), Lane 5: MAP K-10 (hygromycin cassette), Lane 6: *ΔMAP3773c* (*MAP3773c* inner flank), Lane 7: *ΔMAP3773c* (*MAP3773c* outer flank), Lane 8: *ΔMAP3773c* (internal *MAP3773c*), Lane 9: *ΔMAP3773c* (hygromycin cassette). (**c**) Primers used to confirm Δ*MAP3773c*.

Disruption of the *MAP3773c* gene was accomplished by the insertion of a hygromycin marker cassette via a double crossover event between the homologous flanking regions (Fig. 1a). *MAP3773c* deletion was confirmed using polymerase chain reaction (PCR) with internal and flanking primers (Fig. 1c), which validated the presence of the hygromycin cassette and the absence of the *MAP3773c* gene (Fig. 1b).

### Gene expression analysis

**Figure 2:**
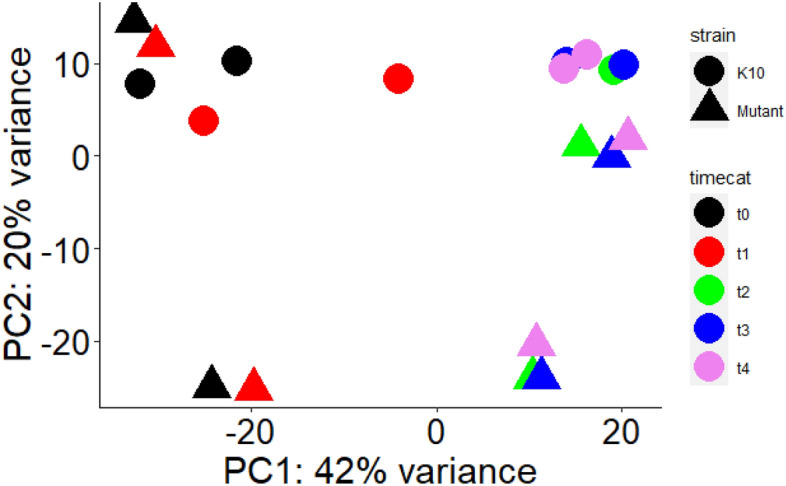
Gene expression analysis. Principal component analysis (PCA) of K-10 and in-frame deletion mutant (Δ*MAP3773c*) by time post-iron restriction. PC1 explains 42% variability and PC2 explains 20% variability among components, strain and time. Iron starvation at different time points is represented by timecat: t0 (control/iron replete), t1 (5 min post-iron starvation), t2 (30 min post iron starvation), t3 (60 min post-iron starvation) and t4 (90 min post-iron starvation) which is represented by different colors. Strain (K10 and Δ*MAP3773c*) is represented by different shapes. The early time points transcripts (left side of graph) are separated from the late time points transcripts (right side of graph).

An average of 16.5 million raw sequencing reads were generated from samples with two replicates from each strain (K-10 and Δ*MAP3773c*) under iron replete and deplete conditions (Supplementary Information 1). Alignment of raw sequence reads against the K-10 genome, showed ~ 90% reads accurately mapped to the reference (Supplementary Information 1). After generating the gene counts, an overall analysis was performed to visualize the general trends in gene expression between parent strain and Δ*MAP3773c*. To understand the variability in our dataset, a principal component analysis (PCA) was conducted. The first principal component (PC1) accounted for 42% variability and second principal component (PC2) accounted for 20% variability in the dataset (Fig. 2). PCA separated the transcripts from early time points post iron starvation from those at later timepoints in PC1 (Fig. 2). At 5 min post-iron restriction (t1), K-10 and *ΔMAP3773c* were clustered together exhibiting similar transcriptional profiles, while at 30 min (t2), 60 min (t3) and 90 min (t4) post-iron restriction, K-10 separated from *ΔMAP3773c* suggesting unique expression profiles associated with both function of *MAP3773c* gene and time of exposure to iron starvation stress.

### Iron stress induces early changes in transcriptional profiles

**Figure 3:**
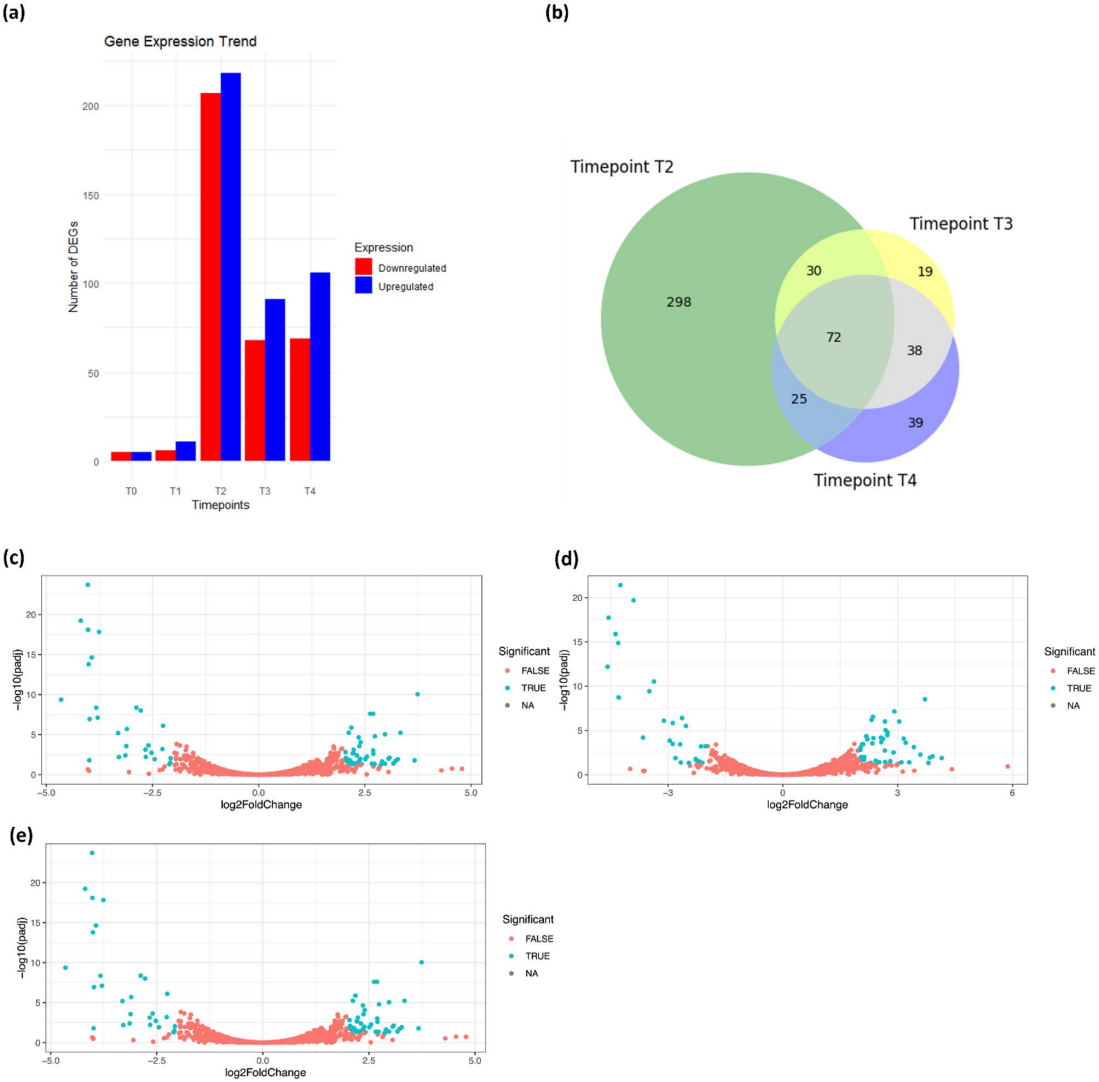
Gene expression profiles under iron stress for Δ*MAP3773c* strain compared to wildtype (K-10). (**a**) Barplot showing the trend of differentially expressed genes (DEGs) by time post-iron restriction for Δ*MAP3773c* vs K-10: T0; control/iron replete, T1; iron starvation post 5 min, T2; iron stress post 30 min, T3; iron starvation post 60 min, T4; iron starvation post 90 min. Only genes that are significant (p < 0.05) are included. (**b**) Venn diagram showing overlap of genes induced by iron stress at time points. Timepoint T2 represents DEGs 30 min post iron-starvation, timepoint T3 represents DEGs 60 min post-iron starvation and timepoint T4 represents DEGs 90 min post-iron starvation. Only genes that are significantly differentially regulated (p < 0.05) are included. (**c**) Volcano plots showing DEGs genes (log2FoldChange) versus – log10(padj) compared Δ*MAP3773c* against K-10 at 30 min. (**d**) Volcano plots showing DEGs genes (log2FoldChange) versus – log10(padj) compared Δ*MAP3773c* against K-10 at 60 min. (**e**) Volcano plots showing DEGs genes (log2FoldChange) versus – log10(padj) compared Δ*MAP3773c* against K-10 at 90 min post-iron stress conditions. The blue dots represents the DEGs in each comparison.

The overall trend of DEGs between K-10 and Δ*MAP3773c* under iron starvation showed a significantly higher number of genes differentially regulated as early as 30 min (Fig. 3) with a tendency towards recovery from iron stress at 60 and 90 min. A total of 425 genes were differentially expressed by Δ*MAP3773c* at 30 min post-iron stress (T2) (Fig. 3a and b) suggesting high sensitivity of Δ*MAP3773c* to the absence of iron in the media. At 30 min post-iron stress 218 genes were upregulated and 207 genes were downregulated (Fig. 3a). The expression of differentially regulated genes at 60 min (T3) and 90 min (T4) post-iron stress decreased to 159 and 175 respectively (Fig. 3a and b).

### Functional analysis of differentially expressed genes by gene enrichment analysis

**Figure 4:**
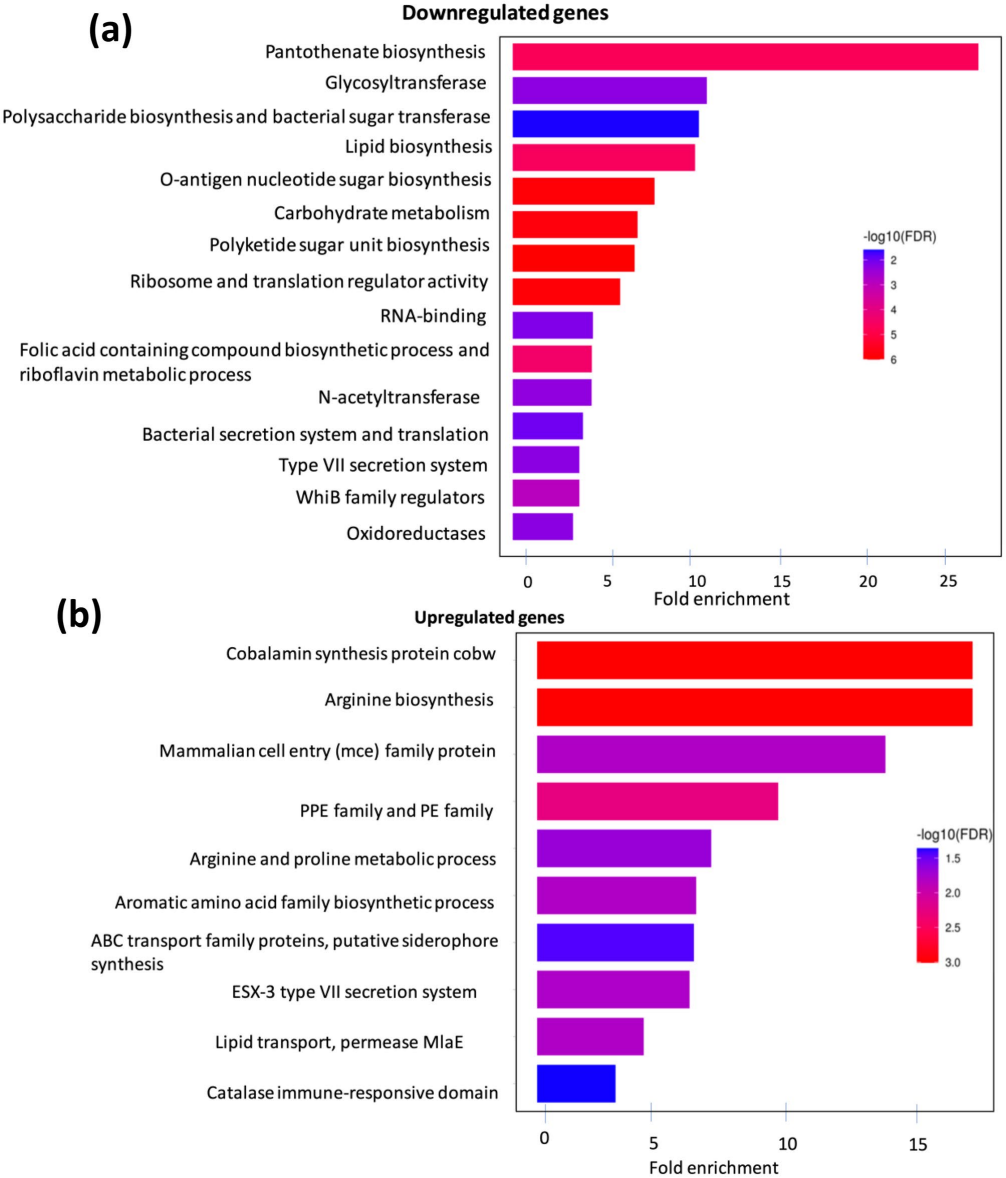
Gene enrichment analysis using differentially expressed genes in Δ*MAP3773c* and K-10. (**a**) Barplot showing downregulated genes that are categorized based on their molecular functions and pathways in Δ*MAP3773c* strain at 30 min post-iron starvation. Top 15 enriched gene sets are shown here. (**b**) Barplot showing upregulated genes that are categorized based on their molecular functions and pathways in Δ*MAP3773c* at 30 min post-iron starvation. Top 10 gene sets are shown here.

Gene enrichment analysis was performed on Δ*MAP3773c* at 30 min post-iron starvation to functionally categorize DEGs based on their molecular functions and pathways. Gene enrichment analysis of DEGs that were downregulated in Δ*MAP3773c* at 30 min post-iron starvation identified pantothenate biosynthesis, polysaccharide biosynthesis, *O*-antigen nucleotide sugar biosynthesis, glycosyltransferase, folic acid-containing compound biosynthetic process and riboflavin metabolic process genes (Fig. 4a). Similarly, the analysis of genes upregulated by Δ*MAP3773c* strain identified cobalamin synthesis, arginine, proline and glutamine biosynthesis, metal transport family, putative siderophore synthesis, mammalian cell entry (MCE), PPE and PE family, putative cholesterol uptake porter CUP1 of MCE4 and among other metabolic pathways at 30 min under iron starvation (Fig. 4b).

### Deletion of *MAP3773c* gene represses CoA biosynthesis pathway

**Figure 5:**
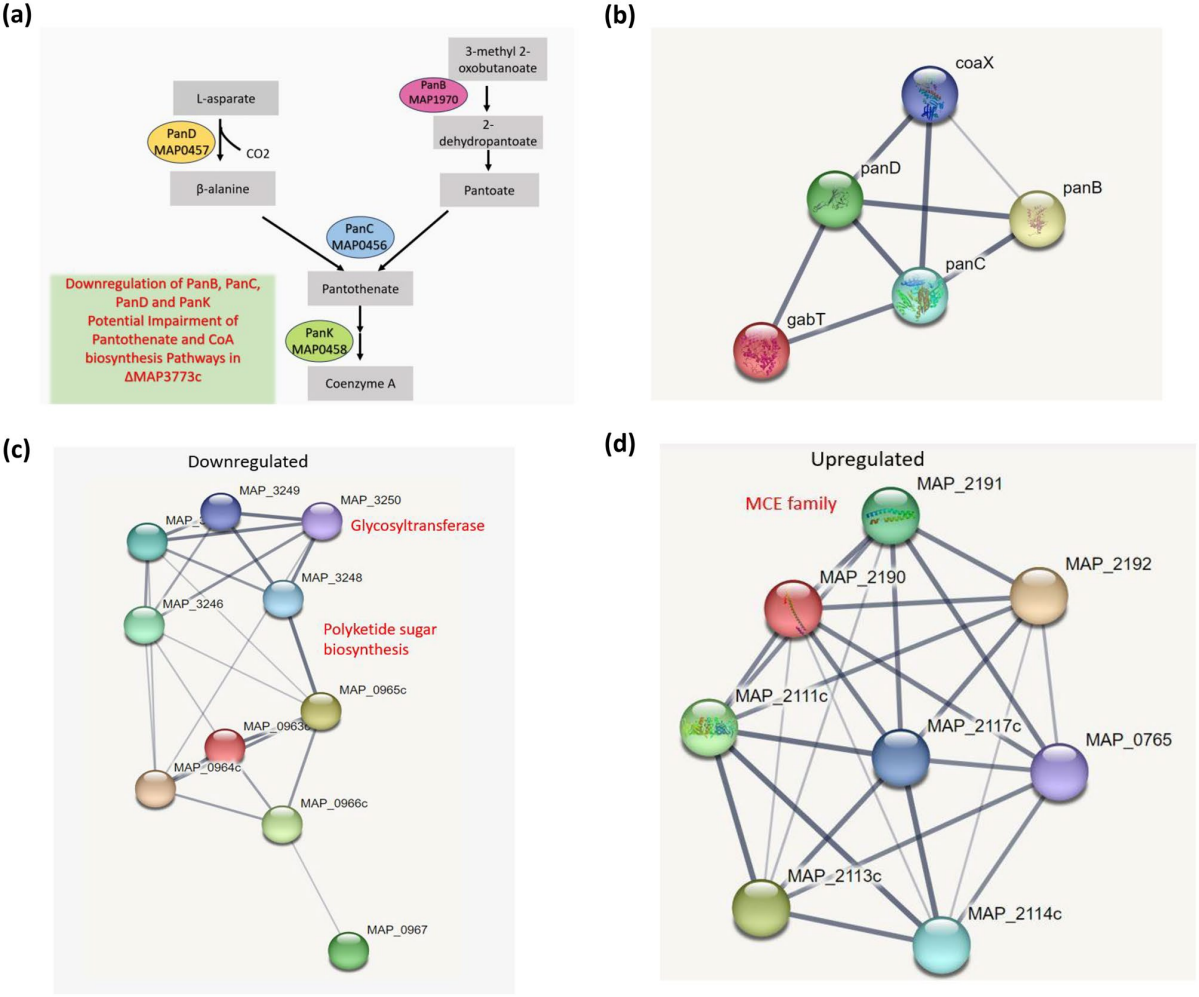
Pantothenate (Pan) and Coenzyme (CoA) biosynthesis pathway in MAP. (**a**) Schematic of genes downregulated in Δ*MAP3773c*. CoA synthesis; *panB* (*MAP1970*), 3-methyl-2-oxobutanoate hydroxymethyltransferase*, panC* (*MAP0456*), pantoate-beta-alanine ligase*, panD* (*MAP0457*), aspartate 1-decarboxylase and *panK* (*MAP0458*), pantothenate kinase. (**b**) Differentially expressed transcripts in Δ*MAP3773c* that are involved in Pan and CoA synthesis were categorized into networks using STRING ver. 11.5. Network analysis showed interaction of six genes *panB, panC, panD*, coax, *MAP0455* and *gabT* (4-aminobutyrate transaminase) for Pantothenate synthesis. Among them *panB, panC, panD*, coax and *MAP_0455* were downregulated in Δ*MAP*3773c strain at 30 min post-iron stress. (**c**) Network analysis of downregulated genes in Δ*MAP3773c* strain at 30 min post-iron starvation involved in glycosyltransferases, polysacaharide and polyketide biosynthesis. Functional interactions between these genes were identified by network analysis. Individual nodes represent proteins. The edges represent the predicted functional associations. The line thickness indicates the strength of the data. (**d**) Network analysis of upregulated genes in Δ*MAP3773c* strain at 30 min post-iron starvation involved in lipid transport. Individual nodes represent proteins with the edges that represent the predicted funcional associations. The line thickness indicates the strength of the data.

CoA is a ubiquitous and essential cofactor involved in regulating several metabolic reactions including production of lipids, formation of cell envelope and virulence factors. *MAP3773c* deletion mutant at 30 min post-iron starvation downregulated Pan and CoA biosynthesis genes. During in-vitro iron stress, MAP can synthesize Pan (pantothenic acid or vitamin B5), the precursor of CoA. The biosynthesis of Pan in MAP is accomplished by *panB* (*MAP1970*), 3-methyl-2-oxobutanoate hydroxymethyltransferase, *panC* (*MAP0456*), pantoate-beta-alanine ligase, *panD* (*MAP0457*), aspartate 1-decarboxylase and *panK* (*MAP0458*), pantothenate kinase. PanK carries out the first step of the CoA biosynthesis pathway. Our data indicates that *MAP3773c* deletion mutant significantly downregulated *panB* (fold change—2.12), *panC* (fold change—2.39)*, panD* (fold change—3.37) and *panK* (fold change—2.77) genes suggesting that Pan and CoA biosynthesis pathway is impaired entirely in the absence of *MAP3773c* gene (Fig. 5a). STRING analysis reveals strong interactions between four genes in the pan operon (*panB*, *panC*, *panD* and *MAP0455*) with coax and *gabT* (4-aminobutyrate transaminase, an enzyme involved in alanine and aspartate metabolism) (Fig. 5b).

### Fatty acid biosynthesis genes and pathways are downregulated in the Δ*MAP3773c* strain

**Figure 6:**
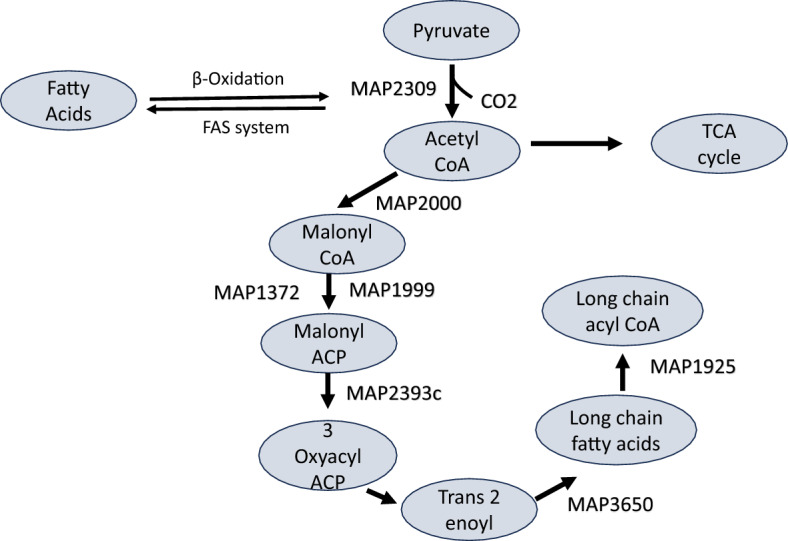
Fatty acids biosynthesis pathway in MAP. Schematic of the fatty acid biosynthesis and elongation by FAS- II to produce mycolic acids. The genes involved in this pathway are shown including *MAP2309c (pdhA),* a pyruvate dehydrogenase*, MAP2000* (acetyl CoA carboxylase), *MAP1999* (3- oxoacyl ACP synthase), *MAP1372* (3-oxoacyl synthase), *MAP1925* (fadD15, CoA ligase), *MAP3650* (enoyl CoA hydratase) all of which are downregulated in Δ*MAP3773c* at 30 min post-iron starvation.

A comparison of Δ*MAP3773c* against K-10 at 30 min post-iron starvation showed downregulated genes with fold change < − 2.0 (Supplementary Information 2) involved in fatty acid biosynthesis, lysine biosynthesis and pyruvate metabolism. KEGG database categorizes the biosynthesis of fatty acids in mycobacteria into two distinct enzyme systems: fatty acid synthase (FAS) I and II. Δ*MAP3773c* downregulated the genes and pathways utilized by MAP to synthesize fatty acids (Fig. 6) which likely impacts the formation of cell wall components, particularly mycolic acids. Genes downregulated in Δ*MAP3773c* compared against those in K-10 were *MAP2309c (pdhA)*, a pyruvate dehydrogenase with fold change of 2.64, *MAP2000* (accD6, acetyl CoA carboxylase) with fold change of 2.60 times, *MAP1999* (kasB_1, ketoacyl-ACP synthase) with fold change 2.52, *MAP1372* (pks11, polyketide synthase) with fold change 2.46, *MAP1925* (fadD15, acyl-CoA synthase) with fold change 11.35 and MAP3650 (enoyl CoA hydratase) with fold change 2.1 (Fig. 6).

### Iron starvation dysregulates cell wall biosynthesis pathway in the Δ*MAP3773c* strain

Δ*MAP3773c* strain at 30 min post-iron starvation downregulated genes involved in the metabolism and biosynthesis of sugar and polysaccharides ≤ 4.0-fold compared against those in K-10 (Supplementary Information 2). The mutant strain downregulated several glycosyltransferase genes including *MAP3246* (glycosyltransferase) with (4.86-fold below K-10 levels)*, MAP3247c* (glycosyltransferase) at 5.06-fold change*, MAP3248* (epimerase family) at a fold change of 6.07*, MAP3249* (carbamoyltransferase)* (*4.86-fold) and *MAP3250* (glycosyltransferase) (7.1-fold). Similarly, two genes *MAP0965c* (epimerase family protein) *and MAP3248* (epimerase family) involved in polyketide sugar unit biosynthesis genes were downregulated at 4.72 and 6.07-fold, respectively (Fig. 5c). Δ*MAP3773c* strain also downregulated *MAP0963c* (oligosaccharide flippase family) (< 2.0 fold)*, MAP0964c* (sugar transferase)*, MAP0966c* (PPE family) and *MAP0967* (transposase) genes at 30 min post-iron starvation (Fig. 5c). The downregulation of these genes in ΔMAP3773c compared against K-10, involved in sugar and polysaccharide metabolism and biosynthesis, as well as specific genes associated with polysaccharide and polyketide sugar unit biosynthesis, indicates an impairment in the synthesis of the essential cellular components of MAP. This impairment likely leads to alterations in cell wall structure, lowered virulence, and dysfunction in the intracellular environment of the host, likely affecting the bacterium's ability to survive and adapt to different environmental conditions.

### Δ*MAP3773c* upregulates various lipid and metal transporters genes in the face of iron starvation

Δ*MAP3773c* strain upregulated genes involved in the transport of cholesterol and lipid at 30 min post-iron starvation ≥ 3.0-fold (Supplementary Information 2). A total of nine genes involved in lipid transport were identified from network analysis (Fig. 5d). MCE genes upregulated by Δ*MAP3773c* are *MAP0765, MAP2111c, MAP2113c*, *MAP2114c*, *MAP2116c, MAP2190, MAP2191, MAP2192,* and *MAP2117c* (ABC transporter permease) which have been proposed to play roles in lipid transport (Fig. 5d).

Δ*MAP3773c* upregulated ATP-binding cassette (ABC) type metal transporters genes*, MAP3774c* (metal ABC transporter permease), *MAP3775c* (ATP-binding cassette domain-containing protein) and *MAP3776c* (zinc ABC transporter substrate-binding protein) at 30 min iron stress. *MAP3173c* (ABC type transporter, mptF) gene was also upregulated. Putative siderophore synthesis genes (*MAP3743-MAP4745)* were also upregulated in Δ*MAP3773c* at 30 min post-iron starvation. Similarly, *MAP0487c, a* putative zinc ABC transporter, transmembrane protein (ZnuB) was upregulated in Δ*MAP3773c* strain at 30 min post-iron starvation. *MAP3737*, a PE family protein expression was upregulated at 30 min post iron starvation in *ΔMAP3773c* strain. *MAP3092*, an iron siderophore ABC transporter substrate-binding protein (FecB) was upregulated at 30 min post-iron starvation. MAP3092 has 99% identity with FecB protein of *Mycobacterium avium* subsp *avium,* which is potentially involved in iron transport ^21^.

### Secretion system and cell envelope genes

Type VII secretion system proteins *MAP3785* (ESX secretion-associated protein EspG)*, MAP3786* (type VII secretion integral membrane protein EccD)*, and MAP3787* (type VII secretion system ESX-3 serine protease mycosin MycP3) were upregulated in Δ*MAP3773c* at 30 min post-iron starvation with fold change ≥ 3.76 (Supplementary Information 2). Additionally, during 30 min of iron starvation, the major facilitator superfamily (MFS) secondary transporters *MAP2516* (MFS transporter)*, MAP0142c* (MFS transporter) and *MAP2441c* (MFS transporter) were upregulated. Among them, two MFS transporters *MAP2516* and *MAP0142* showed sustained upregulation until 60 min of post-iron starvation. This suggests that the bacterium is attempting to increase its capacity to take up any remaining iron in the nutrient deprived environment. Conversely, type VII secretion genes *MAP0160* (WXG100 family type VII secretion target)*, MAP0161* (WXG100 family type VII secretion target) and *MAP1508* (WXG100 family type VII secretion target) were downregulated in Δ*MAP3773c* strain at 30 min post-iron starvation.

At 30 min time following iron starvation, Δ*MAP3773c* upregulated *MAP3750* (MmpS, mycobacterial membrane protein small) and *MAP3751* (MmpL4; mycobacterial membrane protein large) family transporter proteins. However, *MAP1241c* (MmpS family protein) was downregulated at this time point.

### Virulence related and transcriptional regulators genes

Immunological and virulence associated genes include MCE and PE/PPE families of genes. PPE genes that were upregulated in Δ*MAP3773c* at 30 min pos-iron starvation are *MAP1505, MAP1506, MAP1813c, MAP3419c* and *MAP3420c.* The transcription of whiB7 is triggered by both antibiotic treatment and various stress conditions, including heat shock, iron deprivation, and entry into the stationary phase (Geiman et al. ^25^). Δ*MAP3773c* showed a trend toward downregulation of transcriptional regulators belonging to the WhiB family *MAP4273c* (*whiB3*) and *MAP3320* (*whiB1*) at 30-min post iron-restriction. On the other hand, a related transcriptional regulator *MAP3296c* (*whiB*7) was upregulated at the 30 min post-iron starvation.

## Discussion

In this study, a transcriptional analysis was undertaken to define the role of *MAP3773c* in iron sensing and regulation. Further, metabolic pathways regulated by *MAP3773c* under iron replete and deplete conditions were inferred from the transcriptional data. The trend of DEGs over time narrowed our focus to 30 min post-iron starvation (Fig. 3a**)** as the highest number of genes were differentially regulated at that time point.

At 30 min post-iron starvation, Δ*MAP3773c* strain showed downregulation of key metabolic pathways including genes involved in biosynthesis of Pan, glycosyltransferase, polyketide, polysaccharide, folic acid, and riboflavin suggesting that the absence *MAP3773c* impairs synthesis of major cell wall components likely affecting the cell wall biosynthesis and therefore compromising intracellular survival of MAP.

The findings obtained from our RNA-seq analysis were validated through RTqPCR (Supplementary Information 3). Both analyses revealed consistent gene expression profiles across both platforms. As expected, we didn’t observe the same level of gene quantification between RNA-seq and RTqPCR as the latter method accounts for relative quantification while RNA-Seq provides objective and actual reads within each mRNA segment sequenced or absolute quantification.

RNA-sequencing has become the most robust and ubiquitous tool in molecular biology for gene expression profiling at genome wide level. Furthermore, RNA-seq provides absolute quantification in contrast to the reliance on enzymes and housekeeping genes for relative quantification in RT-PCR. Our RNA-seq data had > 98% RNA-Seq coverage at ~ 20 × to ~ 2000× redundancy for all time points and duplicates. With this level of coverage and absolute number of reads with per gene, RNA-seq data provided robust absolute quantification of gene expression in this study quantifying the gene expression level in our study.

CoA cofactor is an essential acyl group carrier indispensable for respiration and lipid metabolism, carbon metabolism as well as in signaling and regulation pathways relying on acetylation-based switches in various organisms ^22^. Under iron starved conditions, Δ*MAP3773c* downregulation of genes involved in Pan and CoA biosynthesis suggest an impairment in respiration, lipid metabolism and carbon metabolism—all crucial for optimal growth and survival of MAP. CoA and Pan initially gained recognition in the field of anti-tuberculosis drug development research when a study highlighted these as potential vaccine candidates ^23^. Sambandamurthy et al. ^23^ observed that *Mtb* mutant lacking Pan (deletion of the *panC* and *panD*), exhibited reduced virulence compared to the wild-type strain. Future experimental infection and cell-invasion studies with ΔMAP3773c are planned to demonstrate the attenuated growth and virulence in Δ*MAP3773c* due to the lack of Pan and CoA biosynthesis pathway.

The absence of *MAP3773c* led to the loss of repression of ABC transporters and putative siderophore synthesis genes (Figs. 4b and 5d). These findings suggest that *MAP3773c* is regulating the expression of metal uptake systems *MAP3774c*-*MAP3776c*, putative siderophore synthesis genes (*MAP3743*-*MAP3755*) and another ABC type transporter (*MAP3731c*) likely compensating for intracellular metal homeostasis.

WhiB family are DNA binding regulators that respond to external conditions such as heat shock, iron depletion, entry into the stationary phase, and exposure to antimicrobial agent stress ^24,25^. They regulate important cellular processes critical to pathogenesis, pathogen survival, and the response to oxidative stress ^26–28^. Δ*MAP3773c* repressed two *whiB* family genes *MAP4273c* (*whiB3*) and *MAP3320* (*whiB1)*. Steyn et al., (2002) ^28^ showed that deletion of *whiB3* resulted in loss of virulence in the guinea pig and attenuation in the mouse model, confirming a role in *Mtb* pathogenesis. *Mtb* WhiB1 is an essential [4Fe-4S] protein that acts as a specific nitric oxide (NO)-sensing DNA-binding protein. The ability of WhiB1 to sense NO and reprogram gene expression likely plays a role in the adaptive responses of *Mtb* to NO generated by the host. Indeed, high concentrations of NO can be lethal to *Mtb* and lower concentrations have been found to facilitate the transition into a latent state ^29–31^. The repression of *whiB* family genes in the absence of the *MAP3773c* also likely influence MAP’s adaptive responses, virulence, and pathogenesis.

MCE proteins are important virulence factors in mycobacteria. They have also been associated with the transport of fatty acids and cholesterol across the impermeable cell envelope of mycobacteria ^32^. Upregulation of MCE genes *MAP2190-MAP2192* and *MAP2113c*-*MAP2114c* in the mutant suggests that these genes are likely regulated by *MAP3773c* since there is loss of repression due to the absence of *MAP3773c* in the mutant strain. The current study, identified expression of genes previously reported by Janagama et al. ^7,33^. The genes that showed upregulation in both studies include cell envelope proteins *MAP3785-MAP3787,* major facilitator family protein *MAP2516,* WhiB7 family transcriptional regulatory protein *MAP3296c,* lipid metabolism *MAP3190* and flavin-utilizing monooxygenase *MAP1084c*. This suggests that these genes in Δ*MAP3773c* are likely regulated by IdeR, a native iron responsive transcription factor*.* In contrast, some genes and pathways including pyruvate metabolism *MAP2309c,* enhanced intracellular survival gene *MAP2325* and integration host factor *MAP1122* are downregulated in the present study but were found to be upregulated underiron starvation in the strains with intact MAP3773c, by Janagama et al. ^7^. The findings indicate that in absence of *MAP3773c*, MAP lacks the regulation of several genes including metabolism, virulence, Fe-S cluster, metal uptake system and secretory system (esx). Future cell invasion assays using Δ*MAP3773c* and complementation of *MAP3773c* in Δ*MAP3773c* strain will help identify whether absence of *MAP3773c* in the genome has any effect in MAP growth and survival.

*Mycobacterium avium* subsp. *paratuberculosis* (MAP) has unique iron requirements for in-vitro growth. Thus, defining iron physiology in MAP is critical in providing a comprehensive understanding of pathogenesis, survival, and persistence of MAP inside the host for an indefinite period. In this study, we showed the regulation of a novel metal regulatory gene *MAP3773c* using genome wide transcriptional analysis. This study demonstrated the direct and indirect roles of *MAP3773c* in response to iron availability. Future studies will focus on complementation of MAP3773c to fully define its function, the effect of deletion on epithelial and monocyte derived macrophage invasion and persistence, as well as *MAP3773c*–IdeR interactions to provide a more comprehensive understanding of iron physiology in MAP under a variety of environmental and cellular conditions.

## Material and methods

### Bacterial strains and culture conditions

MAP K-10 ^34^ and it’s *MAP3773c* deletion mutant (Δ*MAP3773c*) were grown and maintained at 37 °C in Middlebrook 7H9 broth (Fischer Scientific, Inc., Pittsburgh, PA) supplemented with 10% OADC (oleic acid, dextrose, catalase); 0.05% Tween 80 and 2 mg/ml of ferric mycobactin J (Allied Monitor Inc., Fayette, Mo, United States). MAP isolates were determined free of contaminant bacteria by absence of growth on Brain–Heart Infusion (BHI) agar at 37 °C. Minimal medium was prepared as previously described ^35^. Briefly, minimal medium contained 0.5% (wt/vol) asparagine, 0.5% (wt/vol) KH_2_PO_4_ 2% glycerol, 0.5 mg of ZnCl_2_ liter^−1^, 0.1 mg of MnSO_4_ liter^−1^, and 40 mg of MgSO_4_ liter^−1^. Mycobactin J or any source of iron was deliberately excluded from the minimal media to investigate the transcriptional response of MAP under strict iron deficient conditions.

### Construction of deletion mutant of *MAP3773c* (Δ*MAP3773c*)

The deletion of *MAP3773c* gene in MAP K-10 was performed using a site-specific homologous recombination strategy. This deletion mutant was generated using the specialized transducing mycobacteriophage technique developed by Bardarov et al. ^36^. Briefly, the DNA sequence of *MAP3773c* was downloaded from the Kyoto Encyclopedia of Genes and Genomes (KEGG) MAP genome site (http://www.genome.jp/dbget-bin/www_bget?mpa:MAP_3773c) and primers were designed to amplify the upstream region including 84-bp and a downstream region including 111-bp of the gene by using *MAP3773c* UPS (fwd:

5′ GCCTCGGTACCTGCCTCGGTCAATCCGGTAG 3′; rev: 5′ CTCTCTAGAGTTCTCTTGCGCTCGCAGCAC 3′) and *MAP3773c* DWN (fwd: 5′ CTCAAGCTTCGGCTAAGCCGCCAACATCA 3′; rev: 5′ CTCCTCGAGCAAGTAGGTCGGCAATCGTG 3′) primer pairs respectively. The PCR product of *MAP3773c* UPS was cloned into the cosmid vector pYUB854 carrying hygromycin resistance. The PCR product of *MAP3773c* DWN was cloned into the resulting plasmid *MAP3773c* UPS + pYUB854 generating the recombinant cosmid pBUN445. This plasmid was cloned into the conditionally replicating shuttle phasmid vector phAE87 (plasmid form). Then the construct was transfected to *Mycobacterium. smegmatis* mc^2^155 and plated for mycobacteriophage plaques at 30 °C. Phages from a representative plaque were confirmed to be thermosensitive and were amplified into a high titer lysate. This recombinant mycobacteriophage (1 × 10^11^ PFU) was transduced into MAP K-10 (6.4 × 10^9^ CFU) for an MOI = 15.6 and incubated overnight at 39 °C. Transductants were identified on hygromycin agar containing 150 µg/ml after 5 weeks of growth at 37 °C. The double crossover mutant strain was further confirmed using PCR and Sanger sequencing.

## Induction of iron stress

A time course iron restriction analysis was performed to define the transcriptional profiles deployed by MAP with or without *MAP3773c*. Mature cultures of K-10 and *ΔMAP3773c* strains grown in Middlebrook 7H9 supplemented medium were used. The cultured strains were centrifuged at 3500 × g for 10 min at room temperature and the pellets were washed with phosphate-buffered saline (PBS) and a 5 ml aliquot was resuspended in minimal medium and used for iron starvation experiments at different time points. DNase/RNase free plastic containers were used for all experiments. Metal ions from all containers used were chelated by soaking them in 2, 2′ dipyridyl (DIP) for 24 h and autoclaved before use. Similarly, all media were treated with 5% chelex-100 (Bio-Rad) for 24 h with gentle agitation at 4 °C. Chelex-100 resin was removed by filtration through a 0.22-μm filter. Iron starvation was accomplished by treating cultures with DIP (200 μM final) for 5, 30, 60, and 90 min shaken at 200 rpm at 37 °C ^37,38^. Guanidine thiocyanate (GTC) lysis buffer (4 M guanidine thiocyanate, 0.5% sodium N-lauryl sarcosine, 25 mM sodium citrate (pH 7.00) and 0.1 M (beta-mercaptoethanol) was added to each iron starved culture to stabilize the RNA from further transcription and degradation ^39^. Using these thoroughly metal ion depleted labware and media, K-10 and Δ*MAP3773*c, were then exposed to iron restriction for 5, 30, 60, and 90 min in six replicates each. An iron replete baseline culture was also processed for mRNA extraction and sequencing.

### RNA extraction

RNA was extracted from each strain under either iron replete or deplete conditions as described ^39^. Pelleted MAP strains after stabilization with GTC lysis buffer were washed and resuspended in 1 ml PBS + 0.1% Tween80. They were recovered by centrifugation at 3500 × g for 10 min. The pellets were digested with 5 µg/ml lysozyme and incubated for 15 min at room temperature before being lysed in 65 °C Trizol (Life Technologies, Carlsbad, CA, US) using Bead Beater and 0.1 mm silicon beads. Total RNA was isolated from Trizol lysates by adding chloroform followed by centrifugation. The upper aqueous phase was added to a new microcentrifuge tube containing 100% RNase free ethanol which was then processed by Qiagen RNeasy kit DNase treatment and finally with the Ambion Turbo DNA-free kit (Thermo Fisher Scientific, Waltham, MA, US) to remove residual DNA contamination. To ensure RNA integrity, each sample was assessed using the Agilent 5400 Bioanalyzer (Agilent Technologies, Santa Clara, CA, US). RNA samples with an RNA integrity number (RIN) greater than 7 were used for RNA-Sequencing.

### RNA-sequencing

Total RNA extracts from two of the six replicate treatments from each strain (K-10 and *ΔMAP3773c*) were submitted for sequencing (Novogene, Sacramento, CA, USA). RNA-Seq libraries were prepared using the mRNA Seq paired end read library preparation kit and sequenced on NovaSeq 6000 PE150, Illumina platforms. Briefly, removal of ribosomal RNA (rRNA) from total RNA was conducted using Ribo-Zero kit, followed by ethanol precipitation. After the removal of rRNA, mRNA was fragmented, and the first strand cDNA synthesis was carried out using random hexamers. During the second strand cDNA synthesis, dUTPs were replaced with dTTPs in the reaction buffer. The directional library was ready after end repair, A-tailing, adapter ligation, size selection, enzyme digestion, amplification, and purification. The library was then checked with Qubit and real-time PCR for quantification and bioanalyzer for size distribution detection. Quality control (QC) was carried out at each step including sample test, library preparation and sequencing as it can influence the quality of the data. All RNA extracts passed the QC test and were sequenced. The raw sequencing data were received as FASTQ files.

### Transcriptional data analysis

RNA-Seq data analysis was carried out on MSU’s High Performance Computing Center (HPCC) clusters to process sequenced data. First, all reads were checked for QC using FastQC (version 0.11.7) to visualize the quality of the reads and pre-processing was performed using Trimmomatic tool to trim adaptor sequences ^40^. Then, all clean reads were mapped against the reference genome (MAP K-10; Genbank accession number: NC_002944.2) using HISAT2 ^41^. SAMtools was used to sort and index the output files generated by HISAT2 ^42^. Then the indexed bam files were processed using HTSeq which assembles GTF files with gene models and counts mapped reads for each gene to generate count-based matrices. The HTSeq generated count matrices were then used for gene expression normalization and detect differentially expressed genes (DEGs) using DESeq2 ^43^. Differential gene expression analysis was carried out in R 4.3.0 using DESeq2 package ^44^. DESeq2 normalizes gene expression with a “geometric” normalization strategy and Log2 fold changes (LFC) were analyzed by strain (mutant vs K-10) and by time point (0, 5, 30, 60, 90 min). To test whether the LFC attributable to the differences between strains changed across the time course, an interaction term between strain and time point was considered in the design matrix. A likelihood ratio test was used to test the difference between the full model and the reduced model without the interaction term. In addition, DEGs were identified by strain separately for each time point. We used an adaptive shrinkage estimator for the LFC from the apeglm package (https://bioconductor.org/packages/release/bioc/html/apeglm.html). The false discovery rate (FDR) was carried out by adjusting the p-value using the Benjamini–Hochberg algorithm. Genes with expression values with log2 fold change ≥ 1.0 and adjusted p-value ≤ 0.05 or log2 fold change ≤  − 1.0 and adjusted p-value ≤ 0.05 were defined as DEGs. Venn diagram was created using a Python script in Google Colaboratory (a.k.a Colab) (https://colab.research.google.com/) showing distinct and overlapping genes at 30 min post-iron starvation, 60 min post-iron starvation and 90 min post-iron starvation.

### Reverse transcriptase quantitative real time PCR validation

A total of seven genes were selected to perform Reverse Transcriptase Quantitative Real Time PCR (RT-qPCR) to validate the RNA-sequencing results focusing on 30 min post iron starvation. Two step SYBR—green based assay (Applied Biosystems™, Thermo Fisher Scientific, Waltham, MA, US) was performed in QuantStudio™ 6 Pro Real-Time PCR System (Thermo Fisher Scientific, Waltham, MA, US). Primers were designed using web- based tool Primer3Plus https://www.primer3plus.com/index.html and are listed in Supplementary Table S1. The cycle program used was 95 °C for 10 min (activation), 95 °C for 15 s (denaturation) and 60 °C for 1 min repeated for 45 cycles. K-10 was used as the control for analysis. Test and control samples were normalized using two house-keeping genes *secA* and *hsp65*. Relative expression was calculated using 2^−ΔΔCT^ method (7). All samples were conducted in triplicates.

### Gene enrichment and pathways analysis

Functional analysis of the DEGs at 30 min post-iron starvation was performed to identify the set of genes that were involved in several metabolic and cellular processes in both K-10 and *ΔMAP3773c* using ShinyGO 0.77 software ^45^. The categories of genes provided by Gene enrichment analysis were further processed for pathways analysis. Search Tool for the Retrieval of Interacting Genes (STRING) ver. 11.5 ^46^ was used to examine gene networks by uploading MAP gene identification numbers. Functional analysis and pathways were established using KEGG pathways and knowledge reported in the literature including information published on *Mycobacterium tuberculosis* (*Mtb)*.

### Supplementary Information

Supplementary Information 1.

Supplementary Information 2.

Supplementary Information 3.

## Data Availability

The datasets generated during the current study are available in online repositories. The names of the repository/repositories and accession number(s) can be found below: https://www.ncbi.nlm.nih.gov/geo/query/acc.cgi?acc=GSE244734. Enter token exsdocualfkblcl into the box.
