# Supplementary material for: The Fur-like regulatory protein MAP3773c modulates key metabolic pathways in Mycobacterium avium subsp. paratuberculosis under in-vitro iron starvation
Source: Sci Rep. 2024 Apr 18;14:8941. doi: 10.1038/s41598-024-59691-3 (PMC11026511; doi:10.1038/s41598-024-59691-3)
Supplement: Supplementary file 3 — Supplementary Information 3. [file 41598_2024_59691_MOESM3_ESM.docx]

**Supplemental Figure 1**


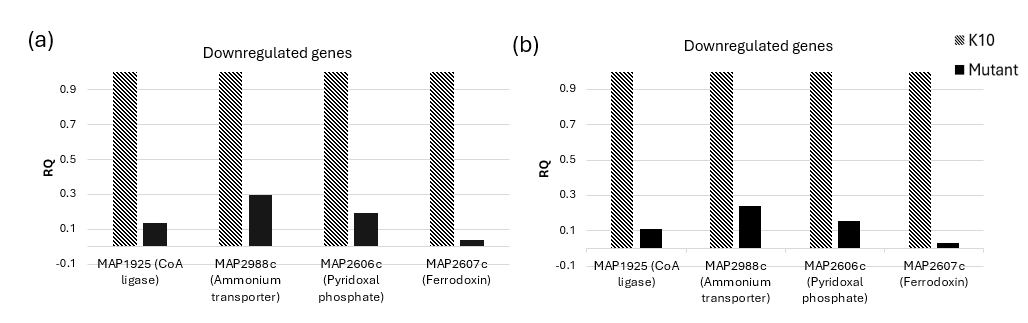


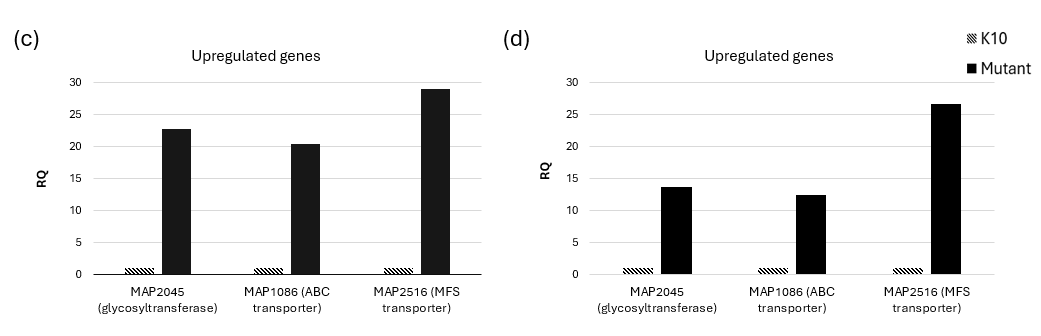


Figure 1. Relative quantification (RQ) values shown by Reverse Transcriptase quantitative PCR (RT-qPCR) of *MAP3773c* deletion strain compared with K10 at 30 minutes post iron starvation. RQ values of four genes shown similar trends in downregulation in the mutant strain in comparison against two housekeeping genes *secA* (**a**) and *hsp65* (**b**), respectively. RQ value of K10 (control) is 1 and the value below 1 represents downregulation. RQ values of three upregulated genes in the mutant strain normalized against two housekeeping genes *secA* (**c**)and *hsp65* (**d**).

Table S1. Primers used in the study to perform RT-qPCR.

| **Gene** | **Primer Sequence (5' - 3')** |
| --- | --- |
| *MAP1925* – CoA ligase | Forward: CATCAAGGACGCCAATCAGG |
|  | Reverse: GTGTCCTCGGTGAAATCGAC |
| *MAP2607c* -ferredoxin family | Forward: CCGTCGAAGCGATCTATTACG |
|  | Reverse: AAGAAGTCGGCGTTGATCTG |
| *MAP2606c*- A pyridoxal phosphate | Forward: ACGAGTGCTATCTGGGATTGG |
|  | Reverse: AAGAAGTCGGCGTTGATCTG |
| *MAP2988c*- Ammonium transporter | Forward: GCGACGTTCGTCTATTTCCC |
|  | Reverse: ACACCGGAATTGATGTGCAC |
| *MAP1086*: ABC transporter | Forward: ACACGACGTTCTTCAATGCC |
|  | Reverse: ACGTCAGGTTGCTTTTCGG |
| *MAP2045*: glycosyltransferase | Forward: GTCGTACAGGTCGCCAATT |
|  | Reverse: AACGATCAGGAAGACCTCGT |
| *MAP2516*: MFS transporter | Forward: GAACCTGGCTTTCATCGTGG |
|  | Reverse: AAGGACAAGGCGAAACAACC |
| *secA* | Forward: GGCCTGCTCCTTGAGGTT |
|  | Reverse: GCGCAAGGTGATCTACGC |
| *hsp65* | Forward: ACATCTCGCTGCTCGGTAAG |
|  | Reverse: GTCGTAGTCGGAGTCGCTGT |
